# Differential Toxicity of Cyanobacteria Isolated from Marine Sponges towards Echinoderms and Crustaceans

**DOI:** 10.3390/toxins10070297

**Published:** 2018-07-18

**Authors:** Ana Regueiras, Sandra Pereira, Maria Sofia Costa, Vitor Vasconcelos

**Affiliations:** 1 CIIMAR/CIMAR, Blue Biotechnology and Ecotoxicology—Centre of Environmental and Marine Research, University of Porto, Terminal de Cruzeiros do Porto de Leixões, Avenida General Norton de Matos, S/N, Matosinhos 4450-208, Portugal; anaregueiras@gmail.com (A.R.); sandra.c.pereira28@gmail.com (S.P.); marysofs@gmail.com (M.S.C.); 2 Department of Biology, Sciences Faculty, University of Porto, Rua do Campo Alegre, Porto 4169-007, Portugal; 3 Faculty of Pharmaceutical Sciences, University of Iceland, Hagi, Hofsvallagata 53, Reykjavik 107, Iceland

**Keywords:** marine cyanobacteria, cyanotoxins, marine sponges, secondary metabolites, marine natural compounds, bioassays, *Artemia salina*, *Paracentrotus lividus*, hemolytic essay

## Abstract

Marine sponges and cyanobacteria have a long history of co-evolution, with documented genome adaptations in cyanobionts. Both organisms are known to produce a wide variety of natural compounds, with only scarce information about novel natural compounds produced by cyanobionts. In the present study, we aimed to address their toxicological potential, isolating cyanobacteria (*n* = 12) from different sponge species from the coast of Portugal (mainland, Azores, and Madeira Islands). After large-scale growth, we obtained both organic and aqueous extracts to perform a series of ecologically-relevant bioassays. In the acute toxicity assay, using nauplii of *Artemia salina*, only organic extracts showed lethality, especially in picocyanobacterial strains. In the bioassay with *Paracentrotus lividus*, both organic and aqueous extracts produced embryogenic toxicity (respectively 58% and 36%), pointing to the presence of compounds that interfere with growth factors on cells. No development of pluteus larvae was observed for the organic extract of the strain *Chroococcales* 6MA13ti, indicating the presence of compounds that affect skeleton formation. In the hemolytic assay, none of the extracts induced red blood cells lysis. Organic extracts, especially from picoplanktonic strains, proved to be the most promising for future bioassay-guided fractionation and compounds isolation. This approach allows us to classify the compounds extracted from the cyanobacteria into effect categories and bioactivity profiles.

## 1. Introduction

Cyanobacteria are photosynthetic prokaryotes, with a high morphological, physiological, and metabolic diversity, with fossil records dating back to 3.5 billion years ago [1]. Secondary metabolite production was essential for their survival allowing for adaptation to several environmental conditions such as variations in temperature, pH, salinity, UV radiation, etc.

Climate change and eutrophication increased the occurrence and frequency of cyanobacterial blooms in water bodies [2], posing human and animals’ health risks due to toxin production. Apart from toxin production, these secondary metabolites have also been shown to be a source of compounds of interest in different industries, such as pharmaceutical, cosmetics, agriculture, energy, etc. In the last decade alone, estimations point to more than 400 new natural compounds extracted from marine cyanobacteria [3]. Coastal water blooms pose another health risk concerning cyanobacterial toxins, as many of them are able to accumulate in both vertebrates and invertebrates [4]. 

Assessing marine cyanobacterial diversity on the Portuguese coast has already been the focus of various studies (e.g., [5,6]), with *Cyanobium*, *Leptolyngbya* and *Pseudanabaena* as the most abundant genera among isolates [6]. Isolated strains from the coast of Portugal were found to be a source of bioactive compounds, both with toxicological and/or pharmaceutical interest [2,7,8,9,10,11,12,13]. Also, Brito, et al. [14] evaluated the potential to produce secondary metabolites for some strains through molecular methods.

In marine environments, cyanobacteria are known to form associations with a variety of invertebrates, such as sponges (Phylum Porifera). Sponges are filter-feeders, capable of filtering thousands of liters of water per day. During this process, some filtered microorganisms can become part of the sponge microbiota. Sponge microbiota diversity can reach up to 4 orders of magnitude, when compared to the one from water column [15]. In temperate ecosystems, it is estimated that 45–60% of sponges have cyanobacterial symbionts (cyanobionts) [16], and are able to cover up to 50% of the sponge cell volume [17]. As they are able to concentrate microorganisms, sponges can be used as a source for cyanobacteria harvesting as already stated by Regueiras, et al. [18]. Sponges are a huge source of bioactive compounds [19], most of them known to be produced by their symbiotic microorganisms [15]. Actinobacteria, Cyanobacteria, Firmicutes, and Proteobacteria (alpha and gamma classes) are the main phyla producing secondary metabolites in sponges [20]. 

Both coccoid and filamentous cyanobacteria have been described in sponges. Recently, Konstantinou, et al. [21] made a review on the diversity of both sponge species harboring cyanobacteria, and cyanobacterial diversity. In Portugal, *Xenococcus*-like and *Acaryochloris* sp. were reported from the intertidal marine sponge *Hymeniacidon perlevis* [22,23]. Regueiras, et al. [18] were also able to identify cyanobacteria belonging to the genera *Synechococcus*, *Cyanobium*, *Synechocystis*, *Nodosilinea*, *Pseudanabaena*, *Phormidesmis*, *Acaryochloris*, and *Prochlorococcus* associated with the same marine sponge. 

Due to a long evolutionary history of both cyanobacteria and marine sponges, co-evolution has already been documented, with some cyanobacteria being passed to new sponge generations through vertical transmission (from sponge to offspring through reproductive cells) [24]. The study of genomes from the symbiotic cyanobacteria “Ca. *Synechococcus spongiarum*” and its comparison with the genome of free-living ones, found adaptations to life inside sponges and the presence of different adaptations in different phylotypes [25,26]. These adaptations may also lead to the production of novel and unique natural compounds.

Bioassay-guided fractionation is a successful strategy in the isolation and discovery of novel compounds [27,28,29,30,31]. To address toxin production, several assays can be used. The use of the brine shrimp *Artemia salina*, has ecological relevance in marine ecosystems, as these organisms are a representation of the zooplankton community and vital on the ecology of seashores [11]. For preliminary toxicity assessment, the brine shrimp lethality assay is a standardized bioassay in marine and aquatic research [32]. For embryogenesis studies, the use of echinoids, such as the sea urchin *Paracentrotus lividus*, is very common. They occupy an important phylogenetic position (deuterostomes) when compared to other invertebrates {Lopes, 2010 #555227}. *P*. *lividus* are also common among the Portuguese seashore and key elements on their habitats [11], capable of producing a great amount of eggs feasible to be fertilized in seawater, and to develop optically clear embryos [33]. Apart from these common assays, less is known on hemolytic toxins from cyanobacteria. Cyanobacterial toxins are able to accumulate in marine vertebrate and invertebrates [34,35], posing risks for mammals, showing the importance of the use of such assays.

The present study aims to do a preliminary assessment on the cyanotoxin potential of marine cyanobacteria isolated from marine sponges. Most studies isolate marine cyanobacteria through filtration of large volumes of water, or by scratching coastal surfaces. In the present study, we aimed to isolate cyanobacteria from marine sponges off the coast of Portugal, as they are able to concentrate microorganisms, allowing them to obtain some cyanobacteria that can be present in seawater in amounts under detection. We intend to evaluate the toxic effects of organic (lipophilic) and aqueous (hydrophilic) crude extracts towards the nauplii of the brine shrimp *A*. *salina* and embryos of the sea urchin *P*. *lividus*, and their hemolytic activity. These assays will be useful to evaluate cyanobacterial potential to produce compounds with relevant bioactivity profiles to be further investigated and possibly identified in the future. This approach allow us to classify the compounds extracted from the cyanobacteria into effect categories and bioactivity profiles.

## 2. Results

### 2.1. Acute Toxicity Assay Using Nauplii of Artemia Salina

**Figure 1:**
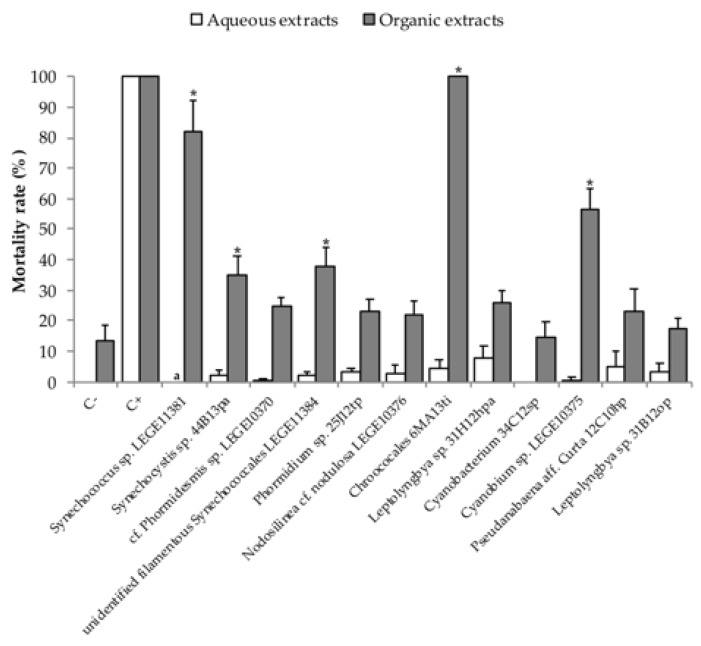
Mortality rate (%) for the *Artemia salina* bioassay, after 48 h of exposure, for the organic and aqueous extracts. Controls used included filtered seawater with 0.1% DMSO for negative control and potassium dichromate (8 μg/mL) for positive control. a Assay not performed; * Statistically significant differences between extract and control.

Aqueous extracts, containing the hydrophilic compounds from the cyanobacterial strains, did not exhibit statistically significant differences against control, in the bioassay to assess mortality in *Artemia salina* nauplii (Figure 1). However, for the organic extracts, toxicity was found after 48 h of exposure. Cyanobacterial strains *Synechoccocus* sp. LEGE11381 (F = 68.80, *p* < 0.000), *Synechocystis* sp. 44B13pa (F = 21.82, *p* < 0.048), unidentified filamentous *Synechococcales* LEGE11384 (F = 24.74, *p* < 0.018), *Chroococales* 6MA13ti (F = 86.73, *p* < 0.000), and *Cyanobium* sp. LEGE10375 (F = 43.50, *p* < 0.000) presented statistically significant differences when compared against the negative control.

### 2.2. Embryo—Larval Acute Toxicity Assay with Paracentrotus Lividus

**Figure 2:**
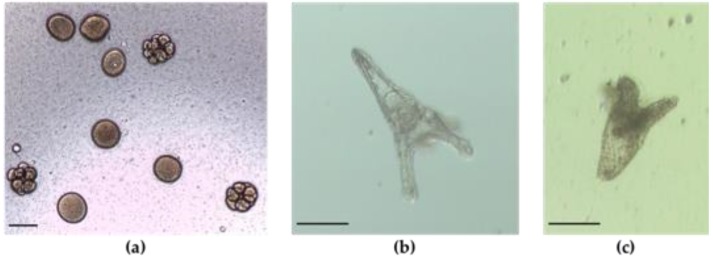
Effects of marine cyanobacterial extracts on embryogenesis of the sea urchin *Paracentrotus lividus*. (**a**) Fertilized sea urchin eggs; (**b**) Normal pluteus larvae resulting from control treatment and (**c**) Abnormally developed larvae resulting from treatments with cyanobacterial extracts. Scale bar: 100 µm.

**Figure 3:**
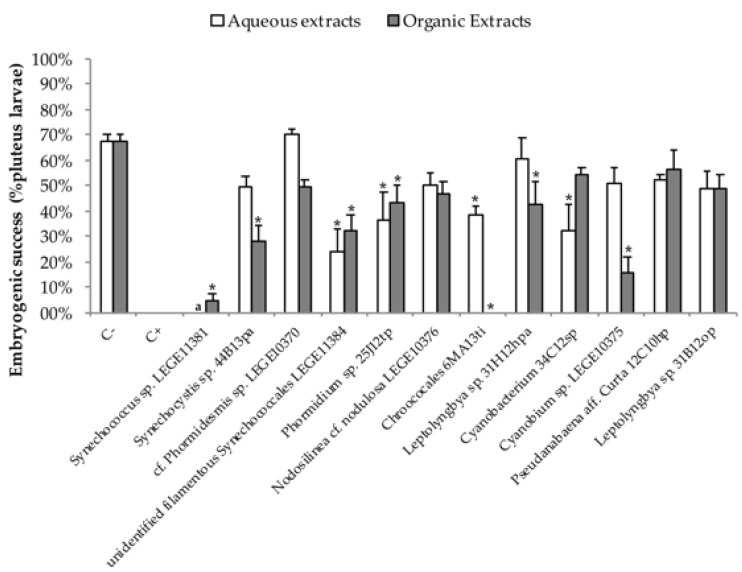
Percentage of pluteus larvae developed (embryogenic success) after exposure to aqueous and organic extracts of the cyanobacterial strains. For the controls, filtered seawater was used with 0.1% DMSO (negative) and potassium dichromate at 4 μg/mL (positive). a Assay not performed; * Statistically significant differences between extract and control.

The toxicity of the cyanobacterial extracts in the bioassay with *P*. *lividus* was determined by analysis of the embryogenic success, i.e., the ability of the fertilized egg to reach the stage of pluteus larvae, and through growth of pluteus larvae (Figure 2). Development arrest indicates that no normal pluteus larvae were produced. The results gathered after 48 h of incubation with cyanobacterial extracts revealed that in the control, 67.5 ± 6.1% of the sea urchin fertilized eggs developed to normal pluteus larvae, with an average length of 330.0 ± 18.8 µm. Figure 3 shows significant difference in the embryogenic development, at *p* < 0.05, for the organic extract of the following strains: *Synechococcus* sp. LEGE11381 (F = −62.78, *p* < 0.000), *Synechocystis* sp. 44B13pa (F = −41.80, *p* < 0.000), unidentified filamentous *Synechococcales* LEGE11384 (F = −36.05, *p* < 0.000), *Phormidium* sp. 25J12tp (F = −27.22, *p* < 0.010), *Leptolyngbya* sp. 31H12hpa (F = 67.48, *p* < 0.048), and *Cyanobium* sp. LEGE10375 (F = −52.38, *p* < 0.000). The organic extract of the strain *Chroococcales* 6MA13ti caused development arrest with none of the larvae reaching the stage of viable pluteus. Amongst the aqueous extracts, unidentified filamentous *Synechococcales* LEGE11384 (F = −41.75, *p* < 0.001), *Phormidium* sp. 25J12tp (F = −28.75, *p* < 0.033), *Chroococcales* 6MA13pi (F = −30.00, *p* < 0.024), and *Cyanobacterium* 34C12sp (F = −39.25, *p* < 0.002) strains presented significant embryogenic effect. Regarding the results from the positive control, only embryos on gastrula stage were found. 

**Figure 4:**
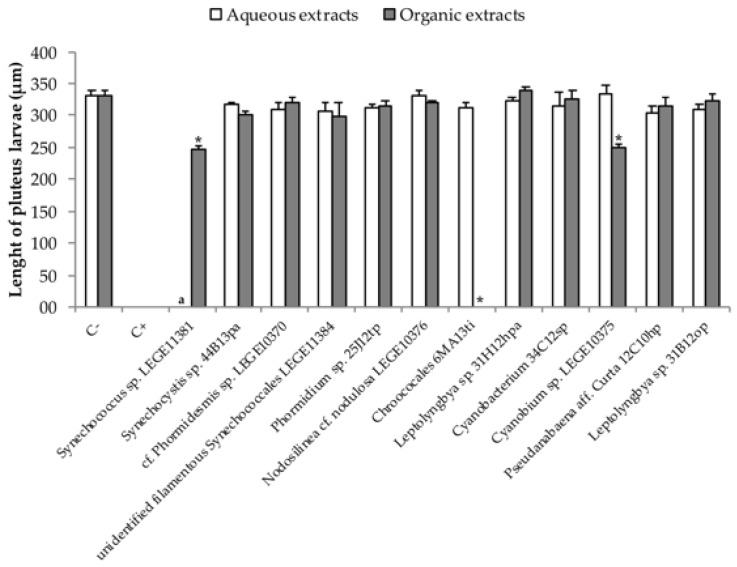
Larval growth from the organic extracts of the cyanobacterial strains. For the controls, filtered seawater was used with 0.1% DMSO (negative) and potassium dichromate at 4 μg/mL (positive). a Assay not performed; * Statistically significant differences between extract and control.

Regarding larval growth data, no significant changes in larval length was observed in the aqueous extracts at *p* < 0.05 [F (11, 36) = 1.039, *p* < 0.434)] (Figure 4). However, differences in larval length were found in organic extracts. These differences were more significant in *Synechococcus* sp. LEGE11381 (246.2 ± 11.5 μm, *p* < 0.001) and *Cyanobium* sp. LEGE10375 (325.7 ± 9.7 μm, *p* < 0.000). 

### 2.3. Hemolytic Assay

The hemolytic activity registered during the assay was below 10%, with the highest value obtained being 7% of activity by the strain *Chroococcales* 6MA13ti, in the organic extract. All strains and extracts did not present significant interference with the hemoglobin content.

## 3. Discussion

To date, most studies exploring the bioactivity of marine cyanobacteria have been focusing on free-living forms. Cyanobacteria can live in association with a variety of marine invertebrates, such as sponges, for example, and it is known that cyanobacteria can affect the biosynthesis of compounds from the host [36] and that symbionts have specific adaptations in their genome [25,26]. The biological potential of associated and/or symbiotic cyanobacteria is still mostly unexplored. In the present study, twelve marine cyanobacterial strains were isolated from sponges of the Portuguese coast. Aqueous and organic crude extracts of the isolated cyanobacterial strains were submitted to ecologically-relevant bioassays in order to do a preliminary assessment on the production of secondary metabolites with relevant bioactivity profiles.

*Artemia* spp. is known for its ability to adapt to different environmental conditions, making it a crucial test organism in ecotoxicology [37]. Results from the bioassay with the brine shrimp *Artemia salina* nauplii did not demonstrate acute toxicity with exposure to the aqueous extracts of the tested cyanobacterial strains. The organic extracts of *Synechococcus* sp. LEGE11381, *Synechocystis* sp. 44B13pa, unidentified filamentous *Synechococcales* LEGE11384, *Chroococcales* 6MA13ti, and *Cyanobium* sp. LEGE10375 cyanobacterial strains proved to be the most toxic to this crustacean species. In contrast with our results, most previous studies with cyanobacteria from the coast of Portugal found aqueous extracts to be more toxic. For example, Leão, et al. [10] reported lethality towards *A*. *salina*, in aqueous extracts in free-living forms from *Nodosilinea*, *Leptolyngbya*, and *Pseudanabaena* genera strains. Also, Frazão, et al. [2] found aqueous extracts of the genera *Cyanobium*, *Synechococcus*, *Leptolyngbya*, *Oscillatoria*, and *Phormidium* more toxic than organic ones. In brackish waters Lopes, et al. [33] also found aqueous extracts more toxic, and organic extracts did not induce more than 7% of mortality on *A*. *salina*. Organic extracts in our work showed a higher toxicity towards *A*. *salina*, leading to an assumption that cyanobacteria associated with marine sponges may produce different metabolites from the ones present in free-living forms of cyanobacteria, and therefore, their toxicological and pharmaceutical potential should be further investigated. The higher values of mortality here observed were all in picocyanobacterial strains. Costa, et al. [9] already reported the potential of these cyanobacteria as a source for novel metabolites. In the present work, toxicity was only found after 48 h. 

In the bioassay with sea urchin *Paracentrotus lividus*, embryogenic toxicity occurred in 58% of the organic extracts and in 36% of the aqueous extracts tested. The unidentified filamentous *Synechococcales* LEGE11384, *Phormidium* sp. 25J12tp, *Chroococcales* 6MA13ti cyanobacterial strains demonstrated embryogenic toxicity in both extracts, which may lead us to infer that, for the same cyanobacterial strain, chemically different bioactive compounds are produced, having the same effect on the embryogenic activity of the sea urchin. Although the *Synechocystis* sp. 44B13pa, unidentified filamentous *Synechococcales* LEGE11384, *Phormidium* sp. 25J12tp, *Leptolyngbya* sp. 31H12hpa, *Chroococcales* 6MA13pi and *Cyanobacterium* 34C12sp cyanobacterial strains have demonstrated to be embryotoxic, no alteration on larval length was observed. This may suggest that the toxicity showed by these cyanobacterial strains only affected the early life stages of the sea urchin embryos development, providing strong evidence for the presence of compounds that interfere with growth factors on cells [11]. The organic extracts of *Synechococcus* sp. LEGE11381 and *Cyanobium* sp. LEGE10375 exhibited interference with the embryogenic development and also with the larval growth. From all the extracts tested, the organic extract from *Chroococcales* 6MA13ti seemed to have the most potent effect on *P*. *lividus* larvae, since it did not allow a normal development of any pluteus larvae. *Cyanobium* sp. organic extracts have already been shown to decrease *P*. *lividus* larvae length [9]. Lopes, et al. [33] found organic extracts from brackish waters to be more toxic to *P*. *lividus*, which is in accordance to our results. The inhibition of larval morphogenesis, here observed, point to the presence of compounds that affect skeleton formation.

Although hemolytic activity has already been documented in strains of *Synechocystis* [38], *Anabaena* [39], *Synechococcus* and *Leptolyngbya* [40], our results showed that in neither organic nor aqueous extracts analyzed, the lysis of the red mammalian blood cells was induced. 

The present study aimed to assess a preliminary cyanotoxicological potential from twelve marine cyanobacteria isolated from the sponges of the Portuguese coast. Eight extracts from cyanobacterial strains have shown a promising potential on the performed ecologically-relevant bioassays (*Synechococcus* sp. LEGE11381, *Synechocystis* sp. 44B13pa; Unidentified filamentous *Synechococcales* LEGE11384; *Phormidium* sp. 25J12tp; *Chroococcales* 6MA13ti; *Leptolyngbya* sp. 31H12hpa; Cyanobacterium 34C12sp; *Cyanobium* sp. LEGE10375). Furthermore, the concentrations of the extracts here used (30 μg mL^−1^) are an ecologically relevant concentration. This emphasizes the premise that sponges can harbor microorganisms with toxicological interest and that these invertebrates can and should be used in order to isolate new cyanobacteria. The extracts with the most promising bioactivity should be further fractionated to identify with more detail the bioactive compounds. Chemical elucidation should be performed once the purest compounds are achieved. 

## 4. Materials and Methods

### 4.1. Cyanobacterial Strains Selection and Biomass Production

**Figure 5:**
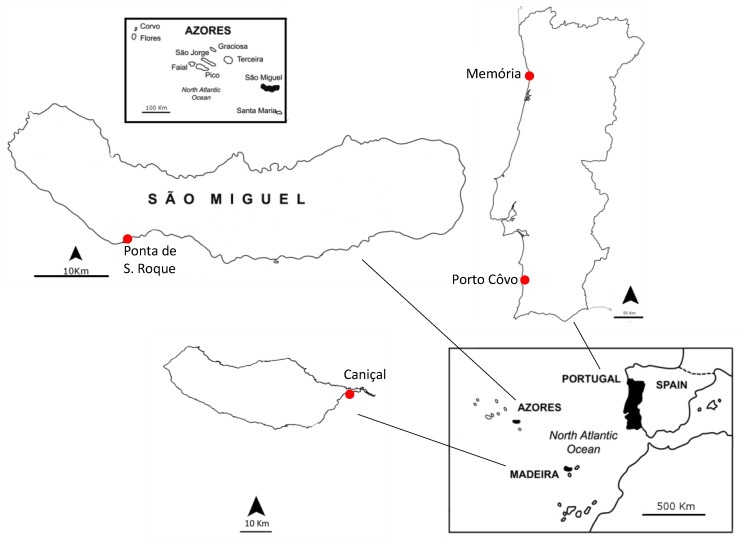
Sampling locations. Two sampling locations were in Portugal mainland: Memória (N 41°13′52.27″, W 8°43′18.34″) and Porto Côvo (N 37°52′3.04″, W 8°47′37.19″). One was in Madeira Island: Caniçal (N 32°44′20.08″, W 16°44′17.55″) and the other in São Miguel Island, Azores: São Roque (N 37°45′15,35″, W 25°38′31.60″).

Cyanobacterial strains used in this study were previously isolated from marine sponges. Marine sponges were collected both from seashore rocks and by scuba diving. A small fraction of sponge tissue was collected in flaks with ambient seawater. Figure 5 shows sampling locations, being all intertidal sites, with exception from the one in Madeira Island, Caniçal (sponges collected through scuba diving). When collected from intertidal areas, beaches were chosen with a combination of sand and rocks. Sponges substratum were rocks or sand. Preparation of sponge samples and cyanobacterial isolation and characterization was done according to Regueiras, et al. [18]. Summarizing, sponges were cleaned of debris and 1 mm of the sponge surface was discarded, using a sterile razor to avoid cultivation of superficial bacteria. Small fragments of the sponge body (<0.5 cm^3^) were placed in 2 different culture media, Z8 liquid media [41], supplemented with 30 g L^−1^ of NaCl and MN liquid medium [42]. Both culture media were supplemented with vitamin B12 and cyclohexamide [42]. After growth, through micromanipulation techniques, as described by Rippka [42], a single cell or filament of cyanobacteria were transfer to new liquid medium, until achievement of unicyanobacterial, non-axenic cultures. 

The selection of cyanobacterial strains was based on growth performance rates and cyanobacterial diversity. Morphological identification followed the criteria of Komárek and Anagnostinis [43,44,45], the Bergey’s manual of systematic bacteriology [46] and Komárek, et al. [47]. Strains are deposited in the LEGE Culture Collection (Ramos et al., 2018). The twelve strains selected (Table 1) were cultured and up-scaled under laboratory conditions at 25 °C, light/dark cycle of 14/10 h and light intensity of approximately 25 × 10^−6^ E/m^−2^s^−1^. After 60 to 90 days of growth, the cyanobacterial biomass produced was collected (through centrifugation or filtration with a 20 μm pore net), frozen at −20 °C and freeze dried. Lyophilized material was kept at −20 °C.

### 4.2. Preparation of Cyanobacterial Extracts

The freeze dried biomass from each cyanobacterial strain was repeatedly extracted with a warm (<40 °C) mixture of dichloromethane and methanol (CH_2_Cl_2_:MeOH) (2:1) (P.A. Sigma, St Louis, MO, USA). Afterwards, the solvents were removed in vacuo and/or under a N_2_ stream. Following the organic extraction, the remaining biomass was subjected to aqueous extraction (ultra-pure water), decanted, and centrifuged at 4600 rpm for 15 min. The resulting supernatant was freeze-dried, weighed, and stored at −20 °C. Just before the tests, organic extracts were dissolved (30 mg mL^−1^) in dimethyl-sulfoxide (DMSO) and aqueous extracts in ultra-pure water.

### 4.3. Bioassays

#### 4.3.1. Acute Toxiciyy Assay Using Nauplii of *Artemia Salina*

In the acute toxicity assay, the nauplii of the crustacean *Artemia salina* were used. The dried cysts (JBL Novotemia, Germany) hatched after 48 h in 35 g/L filtered seawater, at 25 °C, under conditions of continuous illumination and aeration. Toxicity was screened in a 96-well polystyrene plate, with 10–15 nauplii per well and 200 μL of organic or aqueous extract. Filtered seawater with 0.1% DMSO was used as negative control, and potassium dichromate at a concentration of 8 μg/mL as positive control. Four replicates were made for each treatment. The plates were covered with Parafilm to prevent water loss and then incubated at 25 °C, for 48 h in darkness. Dead larvae were counted in each well on an inverted microscope at 24 h and 48 h. Before determining the total number of larvae, organisms were fixed with a few drops of Lugol’s solution. Mortality was calculated through percentage as described by Martins, et al. [11].

#### 4.3.2. Embryo-Larval Acute Toxicity Assay with *Paracentrotus Lividus*

For the embryo-larval acute toxicity assay, sea urchins *Paracentrotus lividus* were captured in the intertidal rocky shore, during low tide in Praia da Memória, Matosinhos, Portugal and immediately transported to the laboratory, in natural sea water and under refrigeration. The protocol employed was the one described by Fernández and Beiras [48]. Briefly, a couple of specimens were dissected, and gametes were collected with a pipette directly from the gonads. The optimal condition from gametes (spherical eggs and mobile sperm) was granted through careful observation under the optical microscope. Eggs were transferred to a 100 mL measuring cylinder containing natural seawater filtered through a 0.45 μm pore filter. A few microliters of sperm were added to the eggs suspension and then carefully stirred to allow fertilization. Fertilized eggs were counted in four 10 μL aliquots in order to determine the fertilization success and egg density. In a 24-well plate, a concentration of 20 fertilized eggs per mL of solution were exposed to organic and aqueous extracts, during 48 h at 20 °C, in darkness. Test solutions consisted of 2.5 mL of each cyanobacterial extract; two negative controls were used, one with only filtered seawater and the other with 0.1% DMSO; as positive control was used potassium dichromate in a concentration of 4 μg/mL. Four replicates were made for each treatment. After 48 h of incubation, the solutions were fixed with 40% formalin. Results were evaluated through percentage of pluteus larvae (embryogenic success) and larval length (larval growth) [11].

#### 4.3.3. Hemolytic Assay

For the hemolytic assay, mice blood, stabilized with heparin, was provided by IBMC Bioterium, from healthy specimens without need to sacrifice the animals. The protocol used was an adaptation of the ones described by Rangel, et al. [49] and Slowing, et al. [50]. Summarizing, the erythrocytes solution was diluted with 30 volumes of a saline solution (0.85% NaCl with 10 mM CaCl_2_) and centrifuged at 1100 *g* for 5 min, discarding the supernatant and then washed three times with the same solution followed by centrifugations (1100 *g* for 5 min). After the final wash, the cells were diluted to a final concentration of 1% in sterile PBS solution. The assay was performed with 100 μL of each extract mixed with equal volume of erythrocytes suspension, using three replicates per treatment. For the negative and positive controls were used PBS and 0.1% Triton100, respectively. Eppendorfs with the mixtures were incubated for 2 h, at a temperature of 37 °C, with slow agitation. After that period, the mixtures were centrifuged at 4000 *g* for 1 min at 4 °C. The supernatants were transferred to a 96 well plate. Hemoglobin content was evaluated spectrophotometrically at 540 nm [49].

(1) $$Hemolytic\;activity=\frac{Abs_{sample}-Abs_{negative\;control}}{Abs_{positive\;control}-Abs_{negative\;control}}\times 100\%\;$$

#### 4.3.4. Analysis

Data collected during the bioassays were analyzed using a one-way analysis of variance (ANOVA), followed by a multi-comparisons Dunnett test (*p* < 0.05). The software IBM SPSS Statistics 24 (Version 24.0.0.0 edition 64-bit, IBM Corporation, New York, NY, USA, 2016) was used for statistical analysis.

## Figures and Tables

**Table 1:** Cyanobacterial strains selected for the present study, with information about the marine sponge it was isolated from and collection site.

Cyanobacterial Strain	Sponge Species	Collection Site
*Synechococcus* sp. LEGE11381	*Polymastia* sp.	Memória
*Synechocystis* sp. 44B13pa	*Polymastia agglutinans*	São Roque, Azores
cf. *Phormidesmis* sp. LEGE10370	*Hymeniacidon perlevis*	Memória
Unidentified filamentous *Synechococcales* LEGE11384	*Phorbas plumosus*	Memória
*Phormidium* sp. 25J12tp	*Tedania pilarriosae*	Memória
*Nodosilinea* cf. *nodulosa* LEGE10376	*Hymeniacidon perlevis*	Porto Côvo
*Chroococcales* 6MA13ti	*Tedania ignis*	São Roque, Azores
*Leptolyngbya* sp. 31H12hpa	*Halichondria panicea*	Memória
*Cyanobacterium* 34C12sp	Unidentified sponge	Caniçal, Madeira
*Cyanobium* sp. LEGE10375	*Hymeniacidon perlevis*	Memória
*Pseudanabaena* aff. *curta* 12C10hp	*Hymeniacidon perlevis*	Memória
*Leptolyngbya* sp. 31B12op	*Ophlitaspongia papila*	Memória
